# Bone transport with the lengthening through the physis in patients having congenital pseudarthrosis of tibia - short-term results

**Published:** 2013-09-25

**Authors:** C Vlad, TS Gavriliu, I Georgescu, D Dan, A Parvan, G Burnei

**Affiliations:** * Maria Sklodowska Curie Emergency Hospital for Children, Bucharest, Romania; ** Floreasca Emergency Hospital, Bucharest, Romania

## Introduction

Congenital pseudarthrosis of tibia (CPT) is still a condition extremely difficult to treat in the field of paediatric orthopaedics. Ilizarov technique, free fibular bone graft, Charnley-Williams method [**1**] are the current treatment options achieving reasonable fusion rate. The solid bone fusion is the most difficult problem to treat. Limb length discrepancy (LLD) is one of the adjacent problems to solve, no matter what method is used to achieve bone fusion. There are three ways to address the LLD: lengthening the short bone, arresting the growth of the normal longer bone or a combination of the two procedures. The “bone transport" [**2**] proposed by Ilizarov combined the procedure of pseudarthrosis site compression with a simultaneous distraction at an adjacent site. The lengthening process during the bone transport may be performed by traction through a proximal tibial osteotomy or through the proximal tibial physis [**3**]. Chondrodiatasis is the term coined by DeBastiani et al. [**3**] and is reserved for the slow traction of the physis. The distraction epiphysiolysis is the term used for the process which encloses a real physis separation [**4**], like a Harris-Salter 1 fracture. The purpose of this paper is to present the results of bone transport with traction through the physis in a case series of patients with CPT, surgically treated in our department.

## Materials and Methods

Eighteen patients with CPT treated in our department between 2001 and 2011 were identified. All the patients were operated in order to achieve solid bone fusion at pseudarthrosis site. The external fixator was used in 11 of 18 cases. We selected only the cases in which the bone transport with distraction epiphysiolysis was performed. All the patient charts and radiograms were reviewed; the data were collected in contingence **Table 1**.

**Table 1:**
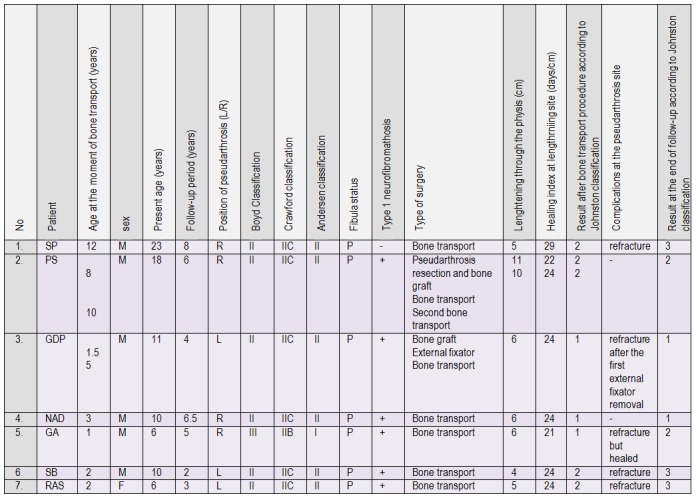
Patients and results

Collected data were: age at the beginning of the study and at the end of follow-up, patient’s sex, side of CPT, classification of pseudarthrosis, the amount of lengthening through the physis, the healing index, complications, classification according Johnston (see **Table 2**) [**5**] after the fixator removal and at the end of study. The treatment respected the principles of Ilizarov external frame application.

**Table 2:** Classification of results according to Johnston

Grade 1	Grade 2	Grade 3
unequivocal union with maintenance of alignment that required no additional surgical treatment. Mild malalignment (<10° in the coronal or sagittal plane) or limb-lengt m-0h discrepancy (<3 cm) expected to require either no treatment or only contralateral epiphyseodesis	equivocal union (residual longitudinal or transverse cortical deficiency) and/or deformity (usually >15° of valgus, procurvatum, or recurvatum) for which additional surgery was required or anticipated	persistent nonunion or refracture that was painful or unstable and required full-time orthotic support.

## Results

**Fig. 1:**
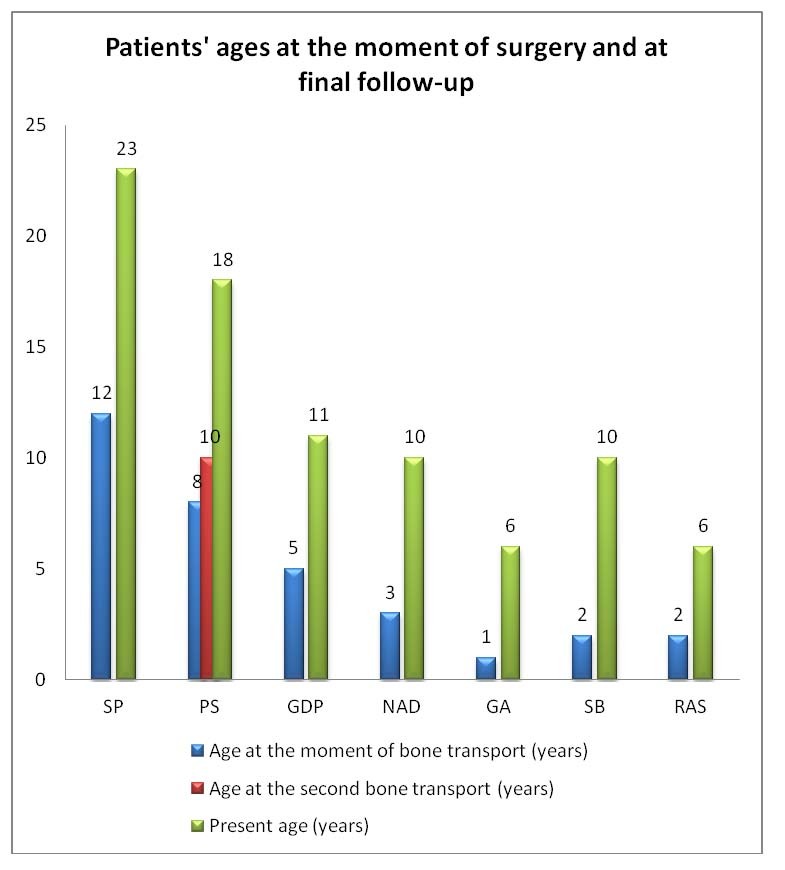
Patients’ ages at the moment of surgery and at final follow-up

Six patients in the study group presented type 1 neurofibromatosis (NF1). All the patients were treated with the bone transport method by using a circular frame. Eight procedures of bone transport were used in seven patients. In one patient, the bone transport was used two times. In one patient the external fixator was previously used without performing a bone transport (patient 3, see **Table 1**). The knee joint was bridged in all cases. The foot was included in the frame for all bone transport procedure. The physis was progressively separated, the distraction rate being of 0.25 mm, four times a day. We were aware of the physeal separation by the sudden decrease of the use of analgesic drugs during the 5th to 10th day of distraction. The separation was also documented with radiograms. 

**Fig. 2:**
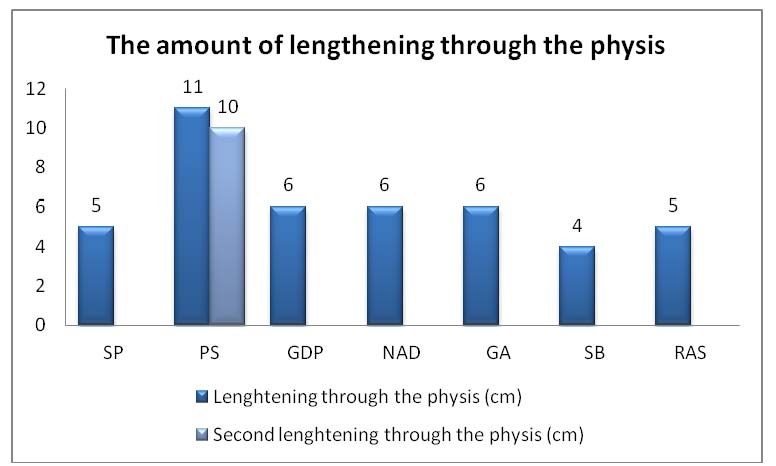
The amount of lengthening through the physis

The lengthening amount through the physis ranged between 4 and 11 cm (**Fig. 2**), average 6.6 cm. 

The new formed bone turned into a good quality solid bone tissue in all cases. The growth process was completely restored in all cases after the fixator removal. The healing index for the physeal distraction site ranged from 21 to 29 days/cm, average 24 days/cm.

 The follow up period after the fixator removal ranged from 2 years to 8 years, averaging 5 years. At the end of treatment, the leg length equality was achieved in all cases. The solid fusion at the pseudarthrosis site was achieved in all cases but fracture recurred in 3 cases, thus, at the end of follow up period, the solid fusion was achieved in only 4 cases. The proximal tibial physis continued to grow in all cases. No major complications were encountered.

Illustrative case report

**Fig. 3:**
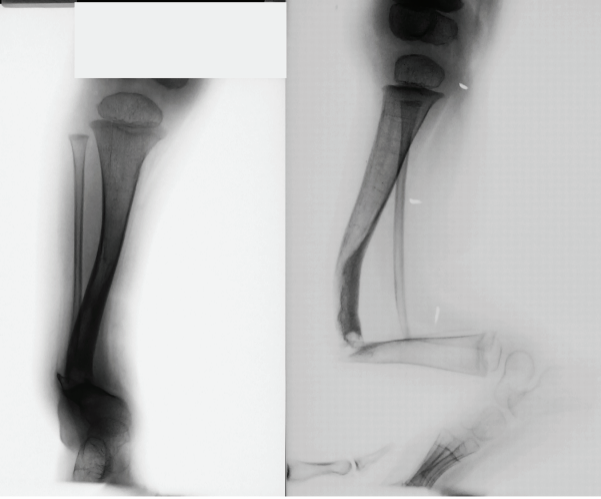
Patient NAD presenting a congenital pseudarthrosis of left tibia, at the age of 2 years

Case 4 (see **Table 1**), is a boy diagnosed during his first year of life with congenital bowing of the right tibia. At the age of 2 years he is addressed to our institution, he presents the fracture of the bowed tibia (**Fig. 3**). 

**Fig. 4:**
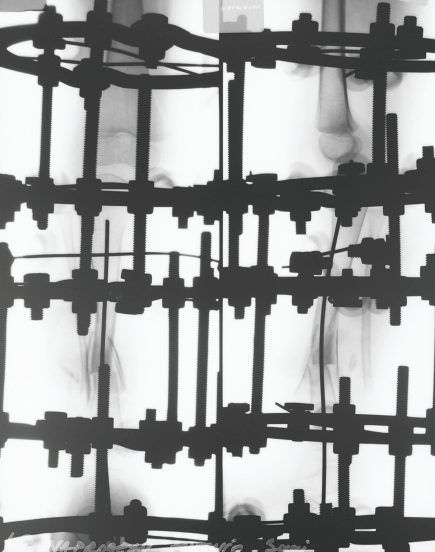
Patient NAD, radiogram of the tibia after the external fixator implantation, the proximal tibial physis is fixated with a ring

The presence of café-au-lait spots and freckling are suggestive elements of type 1 neurofibromatosis. At the age of 2 years and 10 months, the pseudarthrosis was resected, and an Ilizarov external frame was implanted (**Fig. 4**). 

**Fig. 5:**
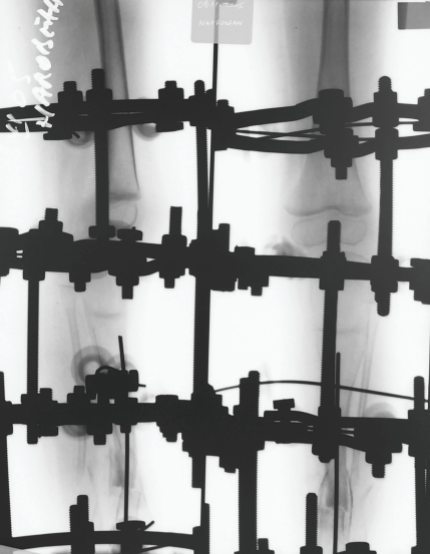
Patient NAD, radiogram of the tibia after 4 cm of traction through the proximal tibial physis

Autogen iliac bone graft was added in the pseudarthrosis site. Five rings composed the external frame: one ring above and one ring below the pseudarthrosis site, one ring at the level of the foot, one ring on the proximal tibial physis, and one ring on the distal femur. The traction through the physis was performed with a rate of 1mm/day achieving 6 cm of bone lengthening (**Fig. 5**). 

**Fig. 6:**
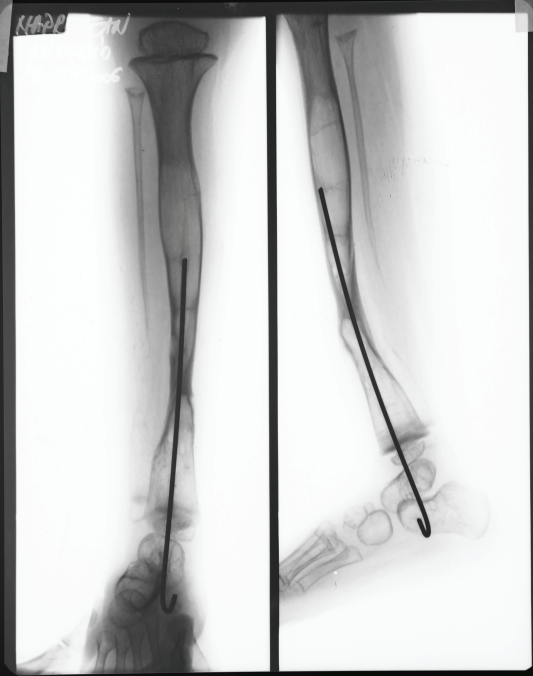
Patient NAD, radiogram of the tibia, 2 years after fixator removal

The pseudarthrosis site was compressed for 10 days with same rate of 1mm/day. The tibia was also splinted with a trans calcaneo talo tibial K-wire. Ten months after the implantation the external fixator was removed (**Fig. 6**). The leg was protected by a knee-ankle-foot orthosis for 1 year.

**Fig. 7:**
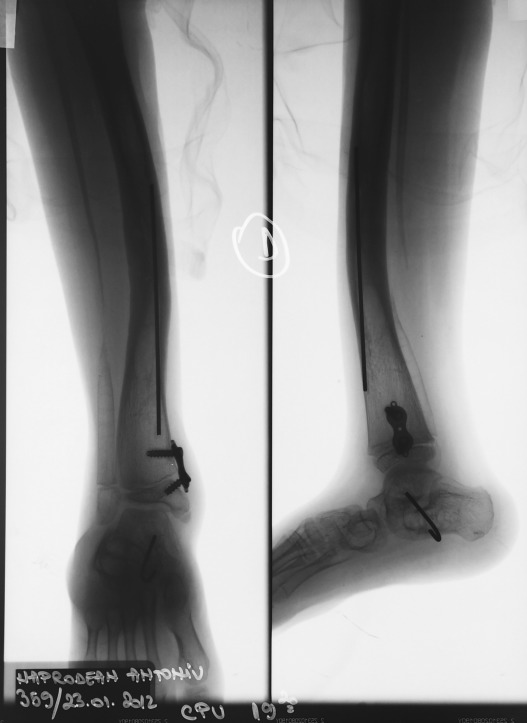
Patient NAD, two years after the distal tibial epiphysiodesis, the tibia is S-shaped but the ankle is well aligned

The pseudarthrosis site continued to heal but the valgus deviation of the ankle forced us to apply an eight plate on the medial side of the distal tibial physis in order to achieve gradual correction. The right alignment of tibia was obtained but the overall aspect of the tibia is S-shaped (**Fig. 7**).

## Discussions

Congenital pseudarthrosis of tibia remains one of the most difficult problems to be treated in the field of paediatric orthopaedics. The surgeon should address not only the pseudarthrosis problem but also the LLD, in order to restore a good functional result. A common problem encountered when dealing with congenital tibial pseudarthrosis is the shortening of the affected tibia. LLD in CPT may easily reach 5 cm, especially when the technique of pseudarthrosis site compression is used. In such circumstances, the only solution is to lengthen the shorter side. Bearing an external fixator is a heavy experience for a child, thus, performing the elongation and the compression in the same time will avoid implanting a second fixator. The process of bone transport was extensively studied by Ilizarov [**6-8**]. The elongation process may be performed through an osteotomy or through a physis. 

 During the 50’s, traction through a physis captured the attention of orthopaedic surgeons. In 1958, Ring [**9**] was the first to report the physeal separation by slow distraction. In 1981, Monticelli and Spinelli [**10**] reported the use of distraction epiphysiolysis in 16 patients. Peltonen experienced the distraction epiphysiolysis in studies on pigs and sheep [**11**]. In 1986, Connolly [**12**] reported the use of epiphyseal traction in animal studies and in clinical practice, but, without success because of early fixator removal. Ilizarov performed extensive research concerning external frame but it was only in 1989 that he reported the effects of tension-stress on genesis and growth of tissues [**7**].

 DeBastiani [**3**] used the physis distraction technique in achondroplastic or short stature patients. Controlled physeal separation was used in tumour surgery. Canadell [**13**] and Betz [**14**] used the acute traction through the physis in the metaphyseal tumour surgery. The method of asymmetrical chondrodiatasis [15-17] was used for the correction of angular deformities.

 In studies on rabbits in 1986, DeBastiani [**18**] made the difference between the distraction epiphysiolysis with true physeal separation, which occurs at a distraction rate of 1mm per day and the chondrodiatasis, with no physeal separation during the slow distraction of 0,25 mm every 12 hours. This process lead to hyperplasia and hypertrophy of the bridging cartilage in the gap of physeal distraction. Hypertrophy and hyperplasia of the growth plate was also described by Sledge in 1976, in animal studies [**19**].

 The physis separation occurs at the proliferative zone of growth plate [**20**]. Studies on rabbits proved that the force developed by distraction device should be around 20-32 Newtons in order to obtain physeal separation [**21**]. In rabbits, this force was obtained by a distraction rate of 0.53 mm/day.

 Hematopoietic cells, fibroblasts and fibres will fill the gap in the distraction zone. From the 7th day, the collagen fibres were organized in the direction of distraction; the new bone formation is evident. Microscopic studies showed the elongated vessels and bone trabeculae oriented in the direction of the elongation [**22**]. In the distracted area, the diameter of bone will increase during distraction, probably because of the increased vascularity in the perichondrial area [**20,23**].

 Complications may accompany the physeal distraction process: bone bowing, subluxations, joint stiffness, fractures [**24**]. Fracture through the base of tibial apophysis may complicate the chondrodiatasis at the proximal tibial level [**25**]. Growth arrest is to be expected in some cases [**25**]. Studies on rabbits proved that the normal growth rate is maintained after physeal distraction [**26**]. Other authors prefer to use this technique near the end of growth in order to minimize the sequels if they appear [**27**]. For Hamanishi et al. [**28**] the use of chondrodiatasis in distal femur was followed by growth arrest especially in patients under 13 years of age.

However, in 1989, Peltonen [**29**] showed that encondral growth continued after physeal distraction in studies on animals. Experimental studies on rabbits showed a protective effect of growth hormone on new bone formation during chondrodiatasis [**30**]. The basic pathology of the CPT is an abnormal growth of the periosteum so one of the concerns in patients having CPT is the consolidation in the distraction site. Some authors warn about the risks of distraction lengthening in patients with CPT presenting dysplasia of proximal tibia [**31**]. We did not find consistent data in our study to support this statement. We performed physeal traction in very young patients, of 4 years old. We found that in the physeal distraction zone the new formed bone was of good quality. We did not encounter any delay in the healing process in the physeal distraction zone. The healing index was between 21 and 29 days/cm, averaging 24 days/cm, which is consistent with other studies [**31**]. In one case, patient 2, see Table 1, we were able to perform the physeal traction two times, at two years interval which proves that the bone transport through the physis does not injure the growth. In none of the cases, the removal of the fixator was not retarded by the healing in the distraction site, but by the healing in the compression site.

 The main limitation of our study is the small number of subjects. Questions remain without answer. One question is if the elongation site does impair the healing process in the compression site. Another question that should be studied on larger groups concerns the healing index, which should be smaller for physeal distraction compared with the osteotomy site distraction.

 In 3 cases of our series the pseudarthrosis site fusion was not achieved at final follow up. Refracture occurred in these 3 patients. Despite the poor quality of periosteal environment, we believe that epiphysiolysis distraction is a valuable method when dealing with LLD in patients with CPT.

**Acknowledgements**

 All patients in the present study were operated by the senior author of this paper, Professor Gh. Burnei

**Sources of Funding**

 This paper is partly supported by the Sectorial Operational Programme Human Resources Development (SOPHRD), financed from the European Social Fund and by the Romanian Government under the contract number POSDRU 64331

**Disclosures**

 None
